# Phosphatases of α-synuclein, LRRK2, and tau: important players in the phosphorylation-dependent pathology of Parkinsonism

**DOI:** 10.3389/fgene.2014.00382

**Published:** 2014-11-07

**Authors:** Jean-Marc Taymans, Veerle Baekelandt

**Affiliations:** Department of Neurosciences, Laboratory for Neurobiology and Gene Therapy, KU LeuvenLeuven, Belgium

**Keywords:** PP1, PP2A, phosphorylation, phosphatase, Parkinson disease, LRRK2, alpha-synuclein, tauopathies, tau proteins

## Abstract

An important challenge in the field of Parkinson’s disease (PD) is to develop disease modifying therapies capable of stalling or even halting disease progression. Coupled to this challenge is the need to identify disease biomarkers, in order to identify pre-symptomatic hallmarks of disease and monitor disease progression. The answer to these challenges lies in the elucidation of the molecular causes underlying PD, for which important leads are disease genes identified in studies investigating the underlying genetic causes of PD. LRRK2 and α-syn have been both linked to familial forms of PD as well as associated to sporadic PD. Another gene, microtubule associated protein tau (MAPT), has been genetically linked to a dominant form of frontotemporal dementia and parkinsonism linked to chromosome 17 (FTDP-17) and genome-wide association studies report a strong association between MAPT and sporadic PD. Interestingly, LRRK2, α-syn, and tau are all phosphorylated proteins, and their phosphorylation patterns are linked to disease. In this review, we provide an overview of the evidence linking LRRK2, α-syn, and tau phosphorylation to PD pathology and focus on studies which have identified phosphatases responsible for dephosphorylation of pathology-related phosphorylations. We also discuss how the LRRK2, α-syn, and tau phosphatases may point to separate or cross-talking pathological pathways in PD. Finally, we will discuss how the study of phosphatases of dominant Parkinsonism proteins opens perspectives for targeting pathological phosphorylation events.

## INTRODUCTION

Parkinson’s disease (PD) is an incurable disease of aging characterized by the progressive death of dopaminergic cells in the midbrain as well as by α-synuclein-rich intracytoplasmic depositions called Lewy bodies (LBs). Treatments which alleviate disease symptoms have been available for several decades; however, these do not halt disease progression. The development of disease-modifying therapies to replace the symptomatic treatments is therefore a major priority in the biomedical research field. Genetic studies of families with a history of PD (genetic linkage studies) as well as of PD patient groups compared to matched groups of healthy individuals (genetic association studies) have identified genes and genomic variants which contribute to the development of PD (Gasser, 2009; Nalls et al., 2011). These studies have revealed at least 20 PD genes, many of which are currently the subject of studies aiming to understand their biology and disease mechanisms. For instance, several PD genes, such as parkin, DJ-1, Pten induced kinase 1 (PINK1), or ATP13A2, contribute to early onset autosomal recessive forms of Parkinsonism. Other genes are linked to Parkinsonism in an autosomal dominant fashion and are responsible for early onset forms of PD (α-synuclein duplications or triplications, some families with mutated α-synuclein) as well as the more common late onset forms of Parkinsonism (α-synuclein mutants, tau, LRRK2, VPS35, or EIF4G1; Houlden and Singleton, 2012). In this review, we focus on the dominant proteins α-synuclein, tau, and LRRK2 in light of the importance of their phosphorylation for their biological functioning.

Mutations in α-synuclein (SNCA, PARK1/4) and mutations in leucine-rich repeat kinase type 2 (LRRK2, PARK8) are linked to autosomal-dominant forms of PD (Gasser, 2009). Also, although protein deposition of microtubule associated protein tau (MAPT) is a feature of Alzheimer’s disease (AD), *MAPT* gene mutations cause frontotemporal dementia (FTD) with Parkinsonism. Interestingly, these three dominant genes in Parkinsonism (MAPT, SNCA, and LRRK2) have also been identified as risk factors for sporadic PD in genome-wide association studies (GWAS; Taymans and Cookson, 2010; Sharma et al., 2012). The dominant mode of disease transmission through these genes also suggests a gain of toxic function mechanism pointing to an inhibition of toxic function as potential therapeutic strategies.

LRRK2, α-syn, and tau are all phosphorylated proteins, and their phosphorylation patterns are linked to disease (Lobbestael et al., 2012; Tenreiro et al., 2014). Early work showed that hyperphosphorylation of tau is correlated to pathology of tauopathies and phosphorylation of α-syn at serine129 is correlated to synucleinopathies (for reviews, see references Martin et al., 2011; Tenreiro et al., 2014); therefore much work has focused on identifying and characterizing kinases of these proteins (for reviews, see references Vancraenenbroeck et al., 2011; Martin et al., 2013b; Tenreiro et al., 2014). The characterization of LRRK2 phosphorylation and the link to disease is still underway although some evidence suggests that a site-dependent mixed phosphorylation state is indicative of disease. Tau and synuclein kinases have been considered as potential therapeutic targets for synucleinopathies and tauopathies and several compounds have been developed for these kinases and tested in preclinical models (for reviews on these topics, see references Vancraenenbroeck et al., 2011; Kramer et al., 2012; Tell and Hilgeroth, 2013). In this review, we will discuss the second main component in the regulation of protein phosphorylation of LRRK2, α-syn, and tau, namely phosphatases. We will briefly introduce the three proteins and discuss what is known about their dephosphorylation and which phosphatases and phosphatase regulators are involved. We will also discuss the relationships between the three proteins with regards to their cognate phosphatases and discuss targeting of phosphatase holoenzymes of LRRK2, α-syn, and tau as a potential phosphomodulatory therapeutic approach.

## ALPHA-SYNUCLEIN

The involvement of α-syn in PD was initially identified through genetic linkage studies in a small number of families (Polymeropoulos et al., 1997), including mutations as well as gene duplications (Chartier-Harlin et al., 2004) and triplications (Singleton et al., 2003). Recently, strong association was shown between α-syn and sporadic PD in GWAS (Satake et al., 2009; Simon-Sanchez et al., 2009). Also, α-syn is a major component of LBs (Spillantini et al., 1997). These arguments illustrate that α-syn is a central player in the pathogenesis of PD.

**FIGURE 1:**
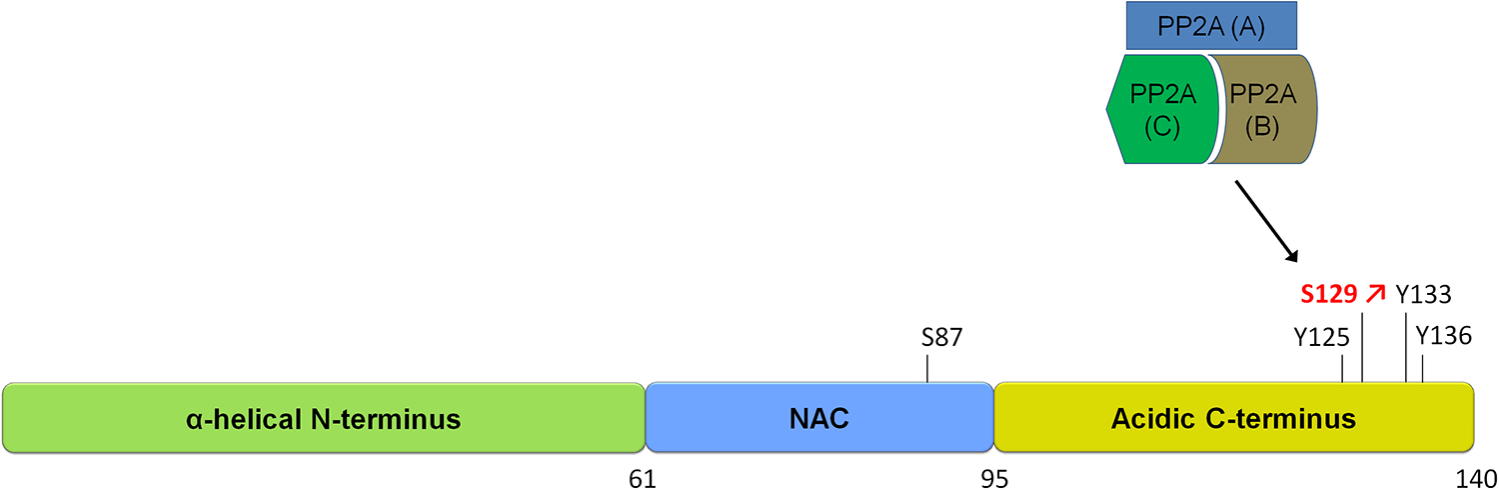
**Schematic of α-synuclein and its phosphorylation sites.** α-syn is a small protein of 140 amino acids in length. It is subdivided into three domains, an N-terminal alpha-helical domain, a central NAC domain (standing for non-Abeta-component) and an acidic C-terminal domain. The S129 site is hyperphosphorylated in disease and is regulated by a phosphatase of the heterotrimeric PP2A class. Please refer to **Table 1** for an overview of studies on phosphatases regulating α-syn phosphorylation.

Studies investigating the phosphorylation of α-syn in diseased and aged brains have shown that α-syn can be phosphorylated at serines (S87, S129) as well as at several tyrosines including Y125, Y133, and Y136 (**Figure 1**). The pY125 modification has been reported to be inversely correlated with PD-related pathology. Indeed, pY125 appears to protect brains against α-syn mediated toxicity, as this modification is reduced in aged human brain tissue and absent in brain tissue affected by Lewy body dementia (Chen and Feany, 2005; Chen et al., 2009). The pS129 modification on the other hand is most often correlated with PD pathology. This notion is primarily supported by the finding that the majority of α-syn in LBs in postmortem PD brains is phosphorylated at S129 (pS129; Fujiwara et al., 2002; Hasegawa et al., 2002; Anderson et al., 2006). The S129 phosphorylation of α-syn in aggregates has also been observed in animal models of PD (Kahle et al., 2000; Neumann et al., 2002; Takahashi et al., 2003). Mechanistic studies have shown that aggregated forms of α-syn are more prone to phosphorylation and that pS129 phosphorylated aggregates accumulate as the disease progresses (Waxman and Giasson, 2008; Mbefo et al., 2010; Paleologou et al., 2010; Waxman and Giasson, 2011), suggesting that the degree of α-syn pS129 phosphorylation is an indicator of disease progression.

The link between S129 phosphorylation and PD pathology has fueled an interest in modulating α-syn phosphorylation at S129 as a potential therapy for PD (Vancraenenbroeck et al., 2011). Multiple kinases have been identified which phosphorylate α-syn at S129, with most evidence pointing to polo-like kinase 2 (PLK2) as the primary phosphorylated of α-syn S129 [for an extended up to date review of α-syn phosphorylation, please refer to Tenreiro et al. (2014)]. A straightforward therapeutic approach based on reducing α-syn phospho-S129 would be to inhibit PLK2 kinase activity; however some contradictory findings should be taken into account. For instance, overexpression of PLK2 in rat brain using adeno-associated viral vectors can suppress α-syn toxicity by promoting autophagy-mediated degradation of phospho-S129 α-syn (Oueslati et al., 2013). Therefore, therapies based on modulating α-syn phospho-S129 appears to require an optimal phosphorylation level rather than a complete dephosphorylation.

## PHOSPHATASES OF α-SYN

Few studies have sought to identify phosphatases of α-syn (Braithwaite et al., 2012); however, concurring data point to PP2A as a major phosphatase of the S129 site. For instance, PP2A enzyme but not PP1 is shown to dephosphorylate α-syn-pS129 *in vitro* (Lee et al., 2011), and treatment of cells with the PP2A inhibitor okadaic acid (OA) but not the PP1 inhibitor tautomycin leads to an increased level of α-syn-pS129. Further characterization showed that α-syn-pS129 was increased upon knockdown of the PP2A catalytic subunit and when PP2A enzyme is methylated.

It is important to note that the phosphatases of the PP2A class function in complexes called holoenzymes, which are composed of regulatory and catalytic phosphatase subunits. In the case of PP2A phosphatases, these are composed of a catalytic subunit (PPP2CA or PPP2CB) together with a scaffold subunit (Aα or Aβ) and a regulatory subunit (of which there are four families, B, B′, B′′, and B^′′′^, each with 2–5 different members). The precise heterotrimeric composition of the holoenzyme guides PP2A to specific substrate sites. Accordingly, the testing of four different holoenzyme compositions shows that holoenzymes with regulatory subunits of the B family are more efficient at dephosphorylating α-syn-pS129 than those of the B′ and B′′ families. Interestingly, α-syn may function in a feedback loop with PP2A, with studies reporting that α-syn has the ability to activate PP2A activity (Peng et al., 2005) and that phospho-S129 α-syn is less efficient at activating PP2A (Lou et al., 2010).

PP2A enzymes have the particularity that their enzymatic activity is positively regulated by its methylation which is itself regulated via the opposing activities of a PP2A-specific methyltransferase and a PP2A-specific methylesterase (PME). Accordingly, treatment of mice with the PME inhibitor eicosanoyl-5-hydroxytryptamide (EHT) increases PP2A methylation as well as decreased α-syn-pS129 levels in brain and a concurrent reduction in synuclein pathology (Lee et al., 2011). Related to this, the diabetes drug metformin was shown to reduce α-syn phosphorylation at S129 through activation of PP2A and inhibition of mammalian target of rapamycin (mTOR; Perez-Revuelta et al., 2014). These studies confirm a primordial role of PP2A in the phosphoregulation of α-syn at S129 and also provide a proof-of-principle that phosphorylation levels of α-syn can be modulated by targeting its phosphatases. Thus far, no phosphatases have been described for the tyrosine phosphorylation sites of α-syn. An overview of α-syn phosphatases is given in **Table 1**.

**Table 1:** Overview of studies reporting phosphatases regulating α-syn phosphorylation.

Site	Identified phosphatase	Comments, references
S129	PP2A	*In vitro* incubation with PP2A [catalytic subunit Cα (PPP2CA), regulatory subunit Bα (PPP2R2A)] dephosphorylates α-syn S129 better than with B′α (PPP2R5A), B′γ (PPP2R5C), or B′′ (PPP2R3A). Knockdown of PPP2CA or PPP2R2A increases α-syn S129 phosphorylation (Lee et al., 2011).
	PPP2R1A	siRNA mediated knockdown of PPP2R1A (Protein phosphatase 2 (formerly 2A), regulatory subunit A, alpha isoform) reported to decrease α-syn S129 phosphorylation levels in neuroblastoma derived cells (Henderson-Smith et al., 2013).
	PTPRA	siRNA mediated knockdown of PPP3R1 (Protein tyrosine phosphatase, receptor type, A) reported to decrease α-syn S129 phosphorylation levels in neuroblastoma derived cells (Henderson-Smith et al., 2013).
	PPP3R1	siRNA mediated knockdown of PPP3R1 (Protein phosphatase 3 (formerly 2B), regulatory subunit B, alpha isoform) found to increase α-syn S129 phosphorylation levels in neuroblastoma derived cells (Henderson-Smith et al., 2013).

*See text for references to reviews discussing α-syn kinases.*

## LRRK2

**FIGURE 2:**
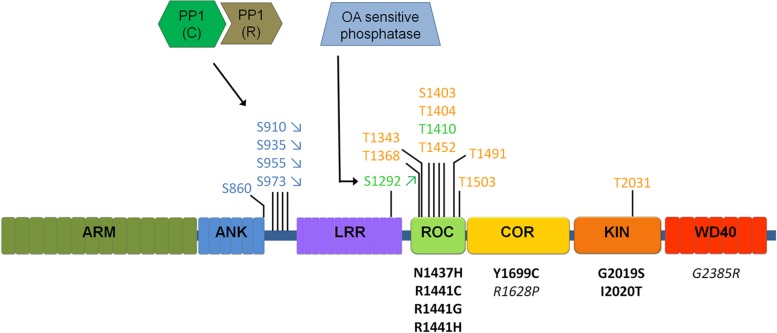
**Schematic of LRRK2 domain structure and currently identified LRRK2 phosphatases.** Phosphorylation sites are indicated above the LRRK2 schematic, for those sites confirmed independently by at least two different groups. Sites represented in blue indicate cellular transphosphorylation sites, in orange *in vitro* autophosphorylation sites and in green autophosphorylation sites confirmed in cells. Disease mutants are indicated below the schematic in black, mutations in italics are risk factor mutations. Arrows (for S910/S935/S955/S973/S1292) indicate the most common regulation of phosphorylation (up or down) observed across most disease mutants. Different sites are likely regulated in different ways, including through PP1 and a yet to be defined phosphatase sensitive to okadaic acid (OA). Please refer to **Table 2** comparing the regulation of phosphorylation at S935 compared to S1292.

LRRK2 is a complex protein of 2527 amino acids containing several predicted functional domains (**Figure 2**). Several arguments underline the importance of LRRK2 for PD. First, LRRK2 is one of the most prevalent causes of monogenic PD. Furthermore, LRRK2 mutations are present in apparently sporadic cases of PD, with prevalences of 2% to up to 40% in certain population groups and LRRK2 was recently genetically associated to PD in several independent GWAS (Satake et al., 2009; Simon-Sanchez et al., 2009). Finally, PD patients carrying the LRRK2 mutations show a clinical and neuropathological profile which is virtually indistinguishable to sporadic PD (Healy et al., 2008), indicating that LRRK2 may contribute to a PD disease pathway common to both familial and sporadic PD.

LRRK2 is a highly phosphorylated protein and phosphosite mapping studies have distinguished two notable clusters of phosphorylation sites, one in or near the Ras of complex proteins (ROC) domain (Greggio et al., 2009; Kamikawaji et al., 2009) and another in the interdomain region between the ankyrin repeat (ANK) and leucine rich repeat (LRR) domains (see **Figure 2**; West et al., 2007; Gloeckner et al., 2010; Nichols et al., 2010; see reference Lobbestael et al., 2012 for a detailed overview of studies reporting LRRK2 phosphorylation sites).

The physiological and pathological relevance of LRRK2 phosphorylation has only just begun to be described. For instance, phosphosites of the ANK-LRR interdomain region, including the S910/S935/S955/S973 sites, are sites phosphorylated by upstream kinases. These sites are detectable in basal conditions in multiple cellular and tissular systems, including for endogenous LRRK2 in Swiss 3T3 or NIH3T3 cells (Nichols et al., 2010; Lobbestael et al., 2013), mouse primary neurons (Lobbestael et al., 2013), mouse brain, kidney, and lung (Deng et al., 2011; Choi et al., 2012; Zhang et al., 2012; Delbroek et al., 2013), mouse embryonic fibroblasts (Dzamko et al., 2012), mouse bone marrow derived macrophages (Dzamko et al., 2012) and human peripheral blood mononuclear cells (Dzamko et al., 2013). The S910 and S935 sites mediate an interaction of LRRK2 with 14-3-3 proteins and regulate LRRK2 cellular localization (Nichols et al., 2010). The search for the kinases responsible for phosphorylating LRRK2 at this cluster is still ongoing. Studies *in vitro* and in COS-7 cells have suggested a role for protein kinase A as an upstream kinase of the S910–S935 sites (Muda et al., 2014), however, these findings are not confirmed in other cell types such as HEK293T cells (Reynolds et al., 2014), suggesting cell-specific mechanisms of phosphorylation. This is further supported by the work of Dzamko et al. (2012) who show that inhibitor of kappa B kinases (IKKs) phosphorylate LRRK2 in bone marrow-derived macrophages upon activation of toll-like receptor signaling which is specific to immune cells. The phosphorylation pattern of LRRK2 is completely different from that of its closest homolog LRRK1 which does not contribute to PD, suggesting that phosphorylation regulation of LRRK2 is a potential mechanism distinguishing LRRK2 from LRRK1 functionally.

Phosphorylation levels of the ANK-LRRK interdomain phosphosites are reduced for several pathogenic mutants such as R1441C/G, Y1699C, I2020T (Nichols et al., 2010; Li et al., 2011; Lobbestael et al., 2013). This observation suggests that the reduction in LRRK2 phosphorylation levels may be involved in the pathogenic mechanism of LRRK2 PD. A corollary of that conclusion is that reduced LRRK2 phosphorylation may be used as a biomarker, however, there are some caveats. For instance, the most prevalent LRRK2 variant in patients, G2019S, does not display reduced phosphorylation levels and a recent study reported no differences in LRRK2 S935 phosphorylation in PBMCs of PD patients compared to matched healthy individuals (Dzamko et al., 2013). Nevertheless, the striking phosphorylation reduction at the ANK-LRR sites seen in all other confirmed LRRK2 pathogenic mutants warrants further evaluation as a disease or diagnostic biomarker.

The other major group of phosphorylation sites for LRRK2 is comprised of autophosphorylation sites. These are sites which were initially identified on LRRK2 protein after *in vitro* incubation with ATP to allow the protein to autophosphorylate itself (Greggio et al., 2009; Kamikawaji et al., 2009; Gloeckner et al., 2010). The majority of these sites cluster in the ROC domain and studies with phospho-mimicking mutants show that at least some of these modifications (T1491D, T1503D) alter LRRK2 GTP-binding properties (Kamikawaji et al., 2009; Webber et al., 2011). The precise physiological relevance of autophosphorylation sites is unknown since the majority of these sites are undetectable in cells, even on overexpressed protein. The notable exceptions are the T1410 site located in the ROC domain identified in overexpressed LRRK2 in HEK293T cells (Pungaliya et al., 2010), the S1058 (Reyniers et al., 2014), and the S1292 site (Gloeckner et al., 2010; Pungaliya et al., 2010; Sheng et al., 2012), located in the 3rd and 13th of the 14 leucine-rich repeats of the leucine-rich repeat domain, respectively, just outside the ROC domain (Vancraenenbroeck et al., 2012). These reports suggest that at least some autophosphorylation events are occurring in cells. Specifically, the S1292 site has been characterized in more detail and displays a number of interesting features. The S1292 site is one of the few autophosphorylation sites located outside ROC. The site is phosphorylated at detectable levels in basal conditions in LRRK2 overexpressed in cell lines or in transgenic mice (Sheng et al., 2012) as well as on endogenous LRRK2 in lymphocytes (Reynolds et al., 2014). In contrast to what is described for the ANK-LRR interdomain sites, the S1292 levels are increased in cells for the majority of pathogenic mutants and decreased in LRRK2 kinase-dead variants (Sheng et al., 2012; Reynolds et al., 2014). Increased phosphorylation levels at S1292 may therefore be indicative of LRRK2’s pathogenic state; however, this remains to be tested in PD patients. Because the kinase activating mutant G2019S shows increased phospho-S1292 and kinase dead mutants show reduced phospho-S1292, it may be suggested that phospho-S1292 levels are indicative of LRRK2 kinase activity in cells, however, some discrepancies appear. For instance, other mutants, such as N1437H, R1441C, or R1441G which are significantly less active in their kinase activity than G2019S nevertheless display similar phospho-S1292 levels relative to the G2019S (Sheng et al., 2012; Reynolds et al., 2014). Two other pathogenic mutants, Y1699C and I2020T, which display kinase activity similar to WT or slightly increased, show varying phospho-S1292 levels depending on the system tested. Indeed, the Y1699C mutant displays increased phospho-S1292 levels in stable overexpression HEK293T cells (Reynolds et al., 2014) and unchanged phospho-S1292 levels in transfected HEK293T cells (Sheng et al., 2012), while the inverse is true for the I2020T mutant. Further work will be required to elucidate the precise regulation of phospho-S1292 levels in LRRK2. It also remains to be shown whether the conclusions here for S1292 (an autophosphorylation site which occurs in cells) also hold true for other autophosphorylation sites or whether this is a new ‘class’ of sites (besides the ROC autophosphorylation cluster and the ANK-LRR interdomain cluster).

Of importance for the development of LRRK2 kinase inhibitors is that the majority of these sites is downregulated by kinase inhibitors and may therefore be used to assess inhibitor activity. For instance, the phosphorylation of autophosphorylation sites such as S1491 is inhibited by LRRK2 kinase inhibitors *in vitro* (Doggett et al., 2012). Also, cellular treatment with kinase inhibitors leads to a dephosphorylation of the ANK-LRR interdomain sites S910/S935/S955/S973 (Dzamko et al., 2010; Deng et al., 2011; Doggett et al., 2012) as well as the S1292 autophosphorylation site (Sheng et al., 2012; Reynolds et al., 2014). Intriguingly, although the ANK-LRR interdomain phosphorylations are not autophosphorylation sites, the observed dephosphorylation of LRRK2 by kinase inhibitors can be attributed to the activity of the inhibitors on LRRK2 itself.

In sum, the emerging picture of LRRK2 phosphorylation is that LRRK2 is a highly phosphorylated protein where at least two, perhaps three, classes of phosphorylation sites can be discerned (see **Figure 2**). A first class of phosphosites in LRRK2 is the *in vitro* autophosphorylation site class. These sites appear after *in vitro* autophosphorylation by purified LRRK2; however, their presence in cells is not confirmed. While these sites offer opportunities to develop assays of LRRK2 kinase activity, their physiological relevance is unclear. Further work will be required to determine whether these sites occur in physiological systems under specific conditions of activation, or whether the appearance of these phosphorylations is an *in vitro* phenomenon. A second class of LRRK2 phosphorylation sites may be termed ‘cellular’ phosphorylation sites, including those sites of the ANK-LRR interdomain region introduced above, exemplified by the S935 site. Finally, the third and most recent class of LRRK2 phosphorylation sites is the class of cellular autophosphorylation sites, exemplified by the S1292 site, which are detected in cells and which also increase after *in vitro* autophosphorylation. The cellular sites (S935) and cellular autophosphorylation sites (S1292) are the most physiologically relevant and the comparison of both sites (summarized in **Table 2**) suggests that these may be useful indicators of LRRK2 activity or pathology. With a few exceptions, pathogenic mutants of LRRK2 display decreased phospho-S935 levels and increased phospho-S1292 levels. It remains to be confirmed whether these changes can be used as diagnostic or disease biomarkers, either individually or together. Interestingly, cellular treatment with kinase inhibitors leads to a reduction of both S935 and S1292. Therefore, both sites are also useful as pharmacodynamic marker to assess activity of kinase inhibitors in cellular and animal models.

**Table 2:** Comparison of the two different LRRK2 phosphorylation sites pS935 and pS1292.

Parameter	pS935	pS1292
Present in cells	YES	YES
Change after *in vitro* autophosphorylation	NO CHANGE	INCREASE
Change after cellular kinase inhibition	DECREASE	DECREASE
N1437H vs. WT	/	HIGHER
R1441C/G vs. WT	LOWER	HIGHER
Y1699C vs. WT	LOWER	SIMILAR
G2019S vs. WT	NO CHANGE	HIGHER
I2020T vs. WT	LOWER	HIGHER
G2385R vs. WT	LOWER	LOWER
Regulation	IKK, PP1, +tbd	AutoP, +tbd
Physiological role	14-3-3 binding, subcellular localization	Putative role in neurite length regulation

*Indicated is the regulation of phosphorylation levels at these sites by autophosphorylation, cellular kinase inhibition, or disease mutants. AutoP, autophosphorylation; tbd, to be discovered.*

## PHOSPHATASES OF LRRK2

We recently reported that protein phosphatase 1 (PP1) is a main phosphatase of the LRRK2 ANK-LRR interdomain sites (Lobbestael et al., 2013). The study first shows that of a panel of recombinant serine/threonine phosphatases, only protein phosphatase 1 can efficiently dephosphorylate LRRK2 *in vitro*. *In vitro* dephosphorylation was demonstrated on purified LRRK2 protein which was previously metabolically labeled by radioactive phosphates, showing that PP1 is responsible for dephosphorylation at the majority of LRRK2’s phosphosites, a finding confirmed for 4 sites with phospho-specific antibodies, i.e., S910, S935, S955, and S973. Upon pharmacological inhibition of cells with either PP1 or PP2A phosphatase inhibitors, it was observed that PP1 but not PP2A inhibition could reverse LRRK2 dephosphorylation.

Interestingly, the effects of PP1 in LRRK2 phosphorylation could be confirmed in multiple cell types including HEK293T, SH-SY5Y neuroblastoma cells, mouse primary cortical neurons, U2OS osteosarcoma cells, NIH3T3 mouse fibroblast cells and A549 human lung cancer cells. This shows that PP1 is active as a LRRK2 phosphatase independent of the cell type tested, and it may be predicted that PP1 can dephosphorylate LRRK2 throughout the body.

Similar to PP2A phosphatases, PP1 class phosphatases are holoenzymes which are composed of one catalytic subunit, responsible for catalyzing the actual dephosphorylation event, and one regulatory subunit, responsible for directing the holoenzyme to its specific substrates. There are more than 150 PP1 regulatory subunits reported, allowing several 100 possible holoenzyme compositions (Bollen et al., 2010). This mode of functioning is necessary given that only three PP1 catalytic subunits are expressed in mammalian cells (PP1α, PP1β, and PP1γ; HGNC codes PPP1CA, PPP1CB, and PPP1CC) which on their own are insufficiently diverse to account for the specificity in the huge volume of phosphatase activity mediated by PP1. Indeed, PP1 together with PP2A (which is represented by only two catalytic subunits, PPP2CA and PPP2CB, see below) account for more than 90% of the protein phosphatase activity in eukaryotes (Moorhead et al., 2007; Virshup and Shenolikar, 2009). This is in stark contrast to the diversity for instance of kinases, of which there are ∼400 serine/threonine kinases (Manning et al., 2002). Therefore, a key issue is to identify the composition of the PP1 holoenzyme by identifying the LRRK2-specific PP1 regulatory subunit which associates with the PP1 catalytic subunit.

There is little data available on the phosphatases involved in the regulation of LRRK2 phosphosites outside of the ANK-LRR interdomain region, however, initial evidence suggests that other phosphatases are at play. Work done at the S1292 site shows that inhibitor induced dephosphorylation of LRRK2 at S1292 is insensitive to the phosphatase inhibitors calyculin A (mixed PP1 and PP2A inhibitor) and OA (selective PP2A inhibitor; Reynolds et al., 2014), in contrast to what is observed at the S935 site where inhibitor induced dephosphorylation is inhibited by calyculin A (Lobbestael et al., 2013). However, the low basal S1292 phosphorylation levels of the R1441G mutant is upregulated by both calyculin A and OA treatment (Reynolds et al., 2014), while the S935 phosphorylation levels of the same mutant is only upregulated by calyculin A (Lobbestael et al., 2013). These findings suggest the hypothesis that PP2A, rather than PP1, is the phosphatase system regulating R1441G LRRK2 at S1292.

## TAU

It may seem surprising to discuss Tau in relation to PD pathogenesis, as this protein has a relatively long history as a protein involved in neurodegenerative dementias; however, accumulating evidence puts this protein to the forefront in PD with a number of reports pointing to specific properties of Tau in PD distinguishing it from Tau in other neurodegenerative disorders. Tau is a microtubule associating protein which is involved in several neurodegenerative diseases including AD, progressive supranuclear palsy (PSP), corticobasal degeneration (CBD), and some cases of frontotemporal lobar dementia (FTLD). Although tau is mostly associated to dementias, the tau gene has also been identified as a risk factor for PD via genome wide association studies (GWAS; Sharma et al., 2012). The genetic association of MAPT locus variants with PD is a striking finding, and is in stark contrast with the fact that no genetic associations of the MAPT locus are observed in AD (Lambert et al., 2013), showing that tau contributes to both AD and PD but via separate mechanisms.

**FIGURE 3:**
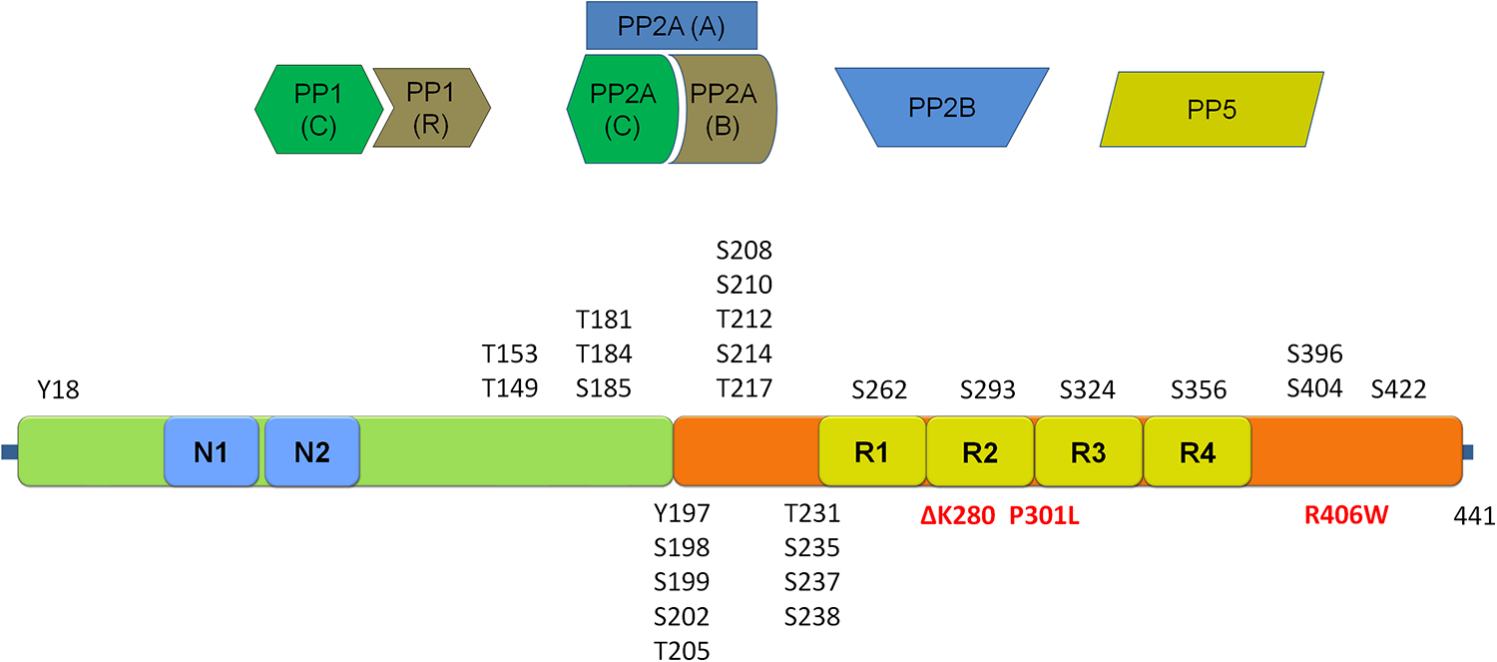
**Schematic representation of the tau protein and its phosphorylation sites.** Depicted in red are mutations associated with frontotemporal dementia and parkinsonism linked to chromosome 17 (FTDP-17). Four main classes of phosphatases reported to regulate tau phosphorylation are given above the schematic. (Tau schematic adapted from Martin et al., 2011 and Tenreiro et al., 2014). Please refer to **Table 3** for an overview of sites regulated by each of the four main tau phosphatase classes.

Tau can occur in six different splice isoforms ranging in size from 352 to 441 amino acids and at least 45 potential tau phosphorylation sites have been reported, including serine, threonine and tyrosine phosphorylation sites [see figure schematic representation of tau protein and the localization of phosphorylation sites, **Figure 3**; for reviews of tau phosphorylation, please refer to Martin et al. (2013a,b) and Tenreiro et al. (2014)]. Several kinases have been reported to phosphorylate tau. These include proline directed kinases [glycogen synthase kinase 3 (GSK3), cyclin dependent kinase 5 (CDK5), and 5′ adenosine monophosphate-activated protein kinase (AMPK)], non-proline directed kinases [casein kinase 1 (CK1), microtubule affinity regulating kinases (MARKs), death associated protein kinase 1 (DAPK1), cyclic AMP-dependent protein kinase A (PKA) and dual specificity tyrosine-phosphorylation regulated kinase 1A (DYRK-1A)] as well tyrosine kinases including Fyn, Abl, and Syk. The inhibition of tau phosphorylation has been proposed as a therapeutic strategy in tauopathies including early phase clinical testing of GSK-3β inhibition (Del Ser et al., 2013).

Tau phosphorylation is important for tau function/dysfunction as hyperphosphorylation of tau is generally correlated with the formation of tau protein aggregates which are major components of neurofibrillary tangles, one of the main neuropathological hallmarks of AD which is also observed in other tauopathies including PD. Phosphorylated tau also more specifically influences its affinity for microtubules and high phosphorylation levels of tau have been reported to negatively influence cytoskeleton, synaptic functions as well as cell viability (Buee et al., 2000). Recently, some work has appeared showing some differences in the tau phosphorylation pattern in post-mortem brain of tauopathies, including PD (Duka et al., 2013). This work revealed notable differences in phosphorylation patterns between PD and other tauopathies. For instance, S202, T205, S262, S409 are hyperphosphorylated in AD and DLB, but are unchanged in PD, while others such as T181, S184, S195, S198, S237, S400 are hyperphosphorylated in all three, AD, DLB, and PD. It remains to be confirmed that these varying phosphorylation patterns are indeed related to the varying pathology observed in these different diseases.

## PHOSPHATASES OF TAU

In light of the importance of tau phosphorylation for its pathology, several studies have sought to identify phosphatases dephosphorylating tau (reviewed in Martin et al., 2013a). These studies reveal a predominant role for PP2A, which is the most efficient phosphatase to dephosphorylate tau *in vitro* at S202, S262, and S356, but not S396 (Bennecib et al., 2000; Kuszczyk et al., 2009; Martin et al., 2009). This finding has been related to AD where to PP2A activity is reduced by up to 50% in AD brains (Voronkov et al., 2011; Martin et al., 2013a), observations pointing towards the potential of targeting PP2A for therapy in tauopathies, although this relation has yet to be explored in PD. Besides the major role for PP2A, other phosphatases have also been found to act on tau phosphorylation. For instance, PP1 has been reported to act on tau at a limited number of sites in AD brains (T212, T217, S262, S396, S422; Rahman et al., 2005). Similarly, PP2B (aka calcineurin) is able to dephosphorylate tau at S262 and S396 (Rahman et al., 2006) while PP5 is reported to dephosphorylate tau at sites S198–S199–S202, T231–S235, S262–S356, S396–S404, and S422 (Gong et al., 2004). An overview of these four classes of phosphatases and the precise sites reported to be regulated by each is given in **Table 3**.

**Table 3:** Overview of different classes of phosphatases reported to regulate tau phosphorylation sites, including PP1, PP2A, PP2B, and PP5.

Site	PP1	PP2A	PP2B	PP5
S198				Gong et al. (2004)
S199	Liu et al. (2005)	Liu et al. (2005)	Saito et al. (1995), Bennecib et al. (2000), Liu et al. (2005), Rahman et al. (2006)	Gong et al. (2004), Liu et al. (2005)
S202	Liu et al. (2005)	Liu et al. (2005), Kuszczyk et al. (2009), Martin et al. (2009)	Saito et al. (1995), Bennecib et al. (2000), Liu et al. (2005), Rahman et al. (2006)	Gong et al. (2004), Liu et al. (2005)
T205	Liu et al. (2005)	Liu et al. (2005), Kuszczyk et al. (2009), Martin et al. (2009)	Saito et al. (1995), Bennecib et al. (2000), Liu et al. (2005), Rahman et al. (2006)	Liu et al. (2005)
T212	Liu et al. (2005), Rahman et al. (2005)	Liu et al. (2005)	Saito et al. (1995), Bennecib et al. (2000), Liu et al. (2005), Rahman et al. (2006)	Liu et al. (2005)
S214	Liu et al. (2005)	Liu et al. (2005)	Saito et al. (1995), Bennecib et al. (2000), Liu et al. (2005), Rahman et al. (2006)	Liu et al. (2005)
T217	Rahman et al. (2005)		Saito et al. (1995), Bennecib et al. (2000), Rahman et al. (2006)	
T231		Saito et al. (1995), Chen et al. (2008)		Gong et al. (2004)
S235	Liu et al. (2005)	Saito et al. (1995), Liu et al. (2005), Chen et al. (2008)	Saito et al. (1995), Bennecib et al. (2000), Liu et al. (2005), Rahman et al. (2006)	Gong et al. (2004), Liu et al. (2005)
S262	Liu et al. (2005), Rahman et al. (2005)	Saito et al. (1995), Liu et al. (2005), Chen et al. (2008), Martin et al. (2009)	Saito et al. (1995), Bennecib et al. (2000), Liu et al. (2005), Rahman et al. (2006)	Gong et al. (2004), Liu et al. (2005)
S293				
S324				
S356		Saito et al. (1995), Chen et al. (2008), Martin et al. (2009)		Gong et al. (2004)
S396	Liu et al. (2005), Rahman et al. (2005)	Liu et al. (2005)	Saito et al. (1995), Bennecib et al. (2000), Liu et al. (2005), Rahman et al. (2006)	Gong et al. (2004), Liu et al. (2005)
S404	Liu et al. (2005)	Saito et al. (1995), Liu et al. (2005), Chen et al. (2008)	Saito et al. (1995), Bennecib et al. (2000), Liu et al. (2005), Rahman et al. (2006)	Gong et al. (2004), Liu et al. (2005)
S409	Liu et al. (2005)	Liu et al. (2005)	Saito et al. (1995), Bennecib et al. (2000), Liu et al. (2005), Rahman et al. (2006)	Liu et al. (2005)
S422	Rahman et al. (2005)		Saito et al. (1995), Bennecib et al. (2000), Rahman et al. (2006)	Gong et al. (2004)

*References of reports showing activity of phosphatases per phosphorylation site mentioned in the first cell of each table row are given in the column of the corresponding phosphatase class. See text for a list of reviews discussing non-phosphatase tau phosphorylation regulators.*

Thus far, the precise regulatory subunits, which may render specificity of these phosphatases to specific tau phosphosites, have yet to be elucidated and confirmed. Some evidence points to the importance of the PP2A regulatory Bα subunit in PP2A mediated phosphoregulation of tau. Expression of the Bα subunit of PP2A is decreased in frontal and temporal cortices of AD brain (Sontag et al., 1999, 2004), although it is not known if Bα subunit expression is altered in PD brain. In *in vitro* assays, the Bα subunit is found to direct the PP2A holoenzyme to microtubules (Xu et al., 2008; Virshup and Shenolikar, 2009), consistent with a role for the Bα subunit in the PP2A holoenzyme dephosphorylating tau.

## POTENTIAL INTERPLAY BETWEEN PHOSPHORYLATION OF LRRK2, α-SYN, AND TAU

As LRRK2, α-syn, and tau are all three involved in dominant Parkinsonism, the hypothesis has been put forward that these three proteins interact in pathological pathways (Taymans and Cookson, 2010), most notably with synuclein and tau acting as toxic proteins and LRRK2 acting as an upstream modulator. Experimental evidence from animal models has begun to support the hypothesis of an interaction between these proteins in PD pathology. For instance, toxicity induced by high α-syn levels in mouse brain is attenuated in LRRK2 knockout mice, both in transgenic mice with high α-syn (inducible CaMKII promoter) expression levels (Lin et al., 2009) as well as after viral delivery of α-syn (Daher et al., 2014), although this has not been replicated in other transgenic mice using other promoters to drive LRRK2 and α-syn expression such as the Thy1 promoter (Herzig et al., 2012) or PrP promoter (Daher et al., 2012) suggesting that the effect is dependent on expression patterns or levels. Several reports show that LRRK2 overexpression affects tau expression or tau phosphorylation (see below), supporting the hypothesis that LRRK2 modulates tau although whether LRRK2 is required for tau toxicity has yet to be tested. Interestingly, the injection of α-syn fibrillar strains into mouse brain is shown to induce tau aggregation (Guo et al., 2013), suggesting an interconnection between α-syn and tau pathological mechanisms. Studies of relationships between LRRK2, α-syn, and tau showing pairwise interactions between these proteins suggest that 3-way interaction studies, which are currently still lacking, are warranted. More information on the overall interplay between these three proteins in PD pathology can be found in recent reviews covering this topic (Greggio et al., 2011; Tenreiro et al., 2014). With regards to the topic of the present review, we highlight below the relationships between LRRK2, α-syn, and tau with regard to their phosphorylation regulation.

As LRRK2 is a kinase, it has been hypothesized that LRRK2 may phosphorylate α-syn or tau. Direct phosphorylation of α-syn by LRRK2 *in vitro* has been tested, however, this led to negative results [reference (Khan et al., 2005) and JMT, VB unpublished results]. Also, in LRRK2 overexpressing mice, phosphorylation levels of α-syn at S129 were found to be unchanged (Herzig et al., 2012) or even reduced compared to controls (Lin et al., 2009), countering the hypothesis that LRRK2 phosphorylates α-syn. Qing and colleagues reported that crude lysates of *E. coli* expressing LRRK2 as source of enzyme could phosphorylate α-syn at S129, suggesting that LRRK2 may regulate α-syn phosphorylation in conjunction with bacterial proteins (Qing et al., 2009), although this is unlikely to be representative of a human physiological situation. The direct phosphorylation of α-syn by LRRK2 can therefore be excluded.

There is, however, evidence that LRRK2 may be involved in regulating tau phosphorylation. First, tau pathology has been observed in post-mortem brain of LRRK2 mutation carriers, including in carriers of the I1371V (Biernacka et al., 2011), N1437H (Puschmann et al., 2012), R1441C (Zimprich et al., 2004), Y1699C (Khan et al., 2005), G2019S (Lin et al., 2010), and I2020T (Ujiie et al., 2012) LRRK2 mutations. Interestingly, genetic studies suggest that tau variants influence LRRK2 disease, more specifically by influencing the age of disease onset (Gan-Or et al., 2012), although another study found that interactions between LRRK2 and tau were at the limit of statistical significance (Biernacka et al., 2011). In cells and *in vivo*, several pieces of evidence point to LRRK2 in regulating tau phosphorylation. MacLeod et al. (2006) found that overexpressed LRRK2 G2019S or I2020T in primary neurons colocalized with phospho-tau punctae in axons. Overexpression of LRRK2 via the ThyI promoter left tau and phospho-tau (S202/T205) levels unchanged in mouse brains (Herzig et al., 2012), although S202 phosphorylation levels were found to be enhanced in brains of BAC transgenic mice expressing LRRK2 G2019S (Melrose et al., 2010). In *Drosophila*, overexpression of LRRK2 G2019S is reported to affect tau dendritic localization and promote tau phosphorylation at T212 through the recruitment of GSK3β (Lin et al., 2010). Interestingly, LRRK2 has been shown to phosphorylate tubulin-associated tau but not free tau (Kawakami et al., 2012). This last finding may be related to the observations in cells that LRRK2 can in certain conditions translocate to skein-like cytoplasmic pools. Although these skein-like cytoplasmic pools have yet to be fully characterized, at least a portion of these are associated to microtubules (Kett et al., 2012). This points to a mechanism whereby LRRK2 may be recruited to microtubules where it may regulate tau phosphorylation with other kinase or phosphatase partners.

In relation to our knowledge of phosphatases of LRRK2, α-syn, and tau and the pathogenic nature of phosphorylations in these proteins, a strategy to target phosphatases in a way that will counteract PD-associated phosphorylation can be proposed. To target LRRK2 disease-related phosphorylations, PP1 would be targeted in order to modulate phosphorylation at its ANK-LRR interdomain phosphorylation sites to a ‘healthy’ level. For instance, pharmacological inhibition of PP1 was shown to increase the S910/S935/S955/S973 phosphorylation to levels comparable to WT. Also, PP1 inhibition was able to reduce the prevalence of LRRK2 presence in skein-like structures observed with several hypophosphorylated disease mutants (Lobbestael et al., 2013). This effect is consistent with a reduction of microtubule associated LRRK2 and therefore reduced risk of LRRK2-mediated tau hyperphosphorylation, suggesting that PP1 inhibition may be a viable therapeutic strategy to inhibit LRRK2 mediated pathology. Thus far, the specific composition of the PP1 holoenzyme targeting the ANK-LRR interdomain sites remains to be elucidated prior to developing LRRK2-specific phosphoregulation strategies. Also, the elucidation of phosphatases regulating other sites, such as S1292, may also reveal other potential phosphatase targets for potential therapies targeting LRRK2 phosphorylation.

For α-syn, targeting PP2A holo-enzymes is a preferred strategy, more specifically through the activation of PP2A to reduce α-syn-S129 phosphorylation levels. The potential of this approach has been shown by pharmacologically enhancing PP2A activity (see above; Lee et al., 2011). The precise PP2A holoenzyme composition for α-syn-S129 has begun to be elucidated, with a preference for the B regulatory subunit above B′ and B′′ subunits. Further elucidation of the preferred C (catalytic) and A (scaffolding) subunits for α-syn-S129 will allow development of phosphatase activation by targeting the PP2A holoenzyme.

For tau, several phosphatases may be targeted, including PP2A, PP1, PP2B, or PP5. The predominant role of PP2A suggests that activation of PP2A may also be a beneficial therapeutic strategy. One issue is whether separate PP2A holoenzymes are responsible for dephosphorylation of tau at the separate phosphosites. Specifically for PD, it may be expected that dephosphorylation of tau at PD hyperphosphorylation sites should be targeted prioritarily, including T181, S184, S195, S198, S237, S400 (Duka et al., 2013). Further research will be needed to describe the PD-tau phosphorylations and their regulation.

## TARGETING OF PHOSPHATASES

Modulation of phosphorylation levels of disease proteins is an attractive approach to develop disease modifying therapies. For instance, much effort has been spent on targeting kinases responsible for pathogenic phosphorylations. Kinases are also attractive drug targets. Indeed, attrition rates during development of drugs acting on kinases are lower than for most other classes of drugs (Walker and Newell, 2009). An important factor to bear in mind with pharmacological inhibition of kinases is that this will influence the phosphorylation levels of all of the kinase’s substrates, both in the disease protein as well as for all of its other substrates. Therefore, for key phosphorylations, it may be necessary to target phosphorylations through other means than by targeting kinases. One possibility is to target phosphatases.

**FIGURE 4:**
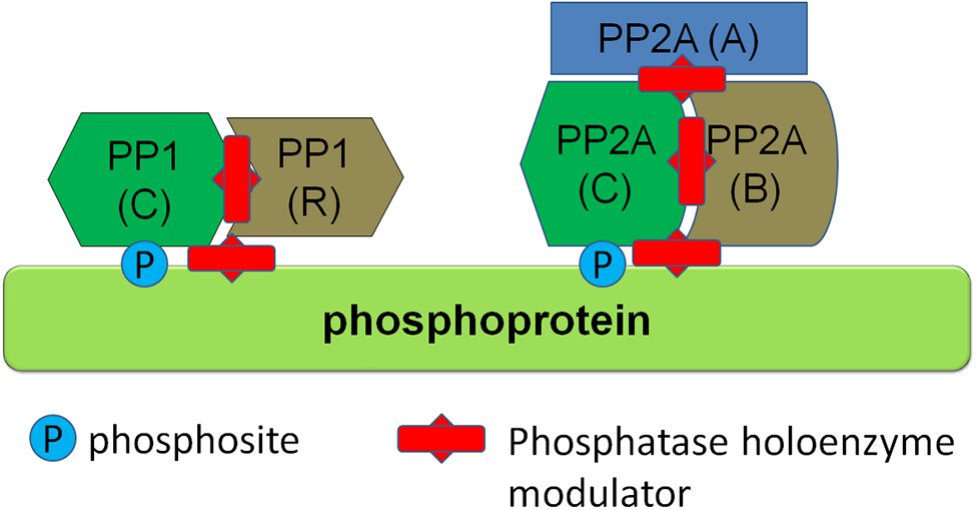
**Regulation of protein phosphorylation via modulators of phosphatase holoenzymes.** Several phosphatase classes, such as PP1 and PP2A class phosphatases depicted here function as multiprotein holoenzymes including a catalytic subunit and regulatory subunits. Phosphatase activity can be modulated by protein-protein interaction modulators (depicted here as red studded bars) which can either disrupt the holoenzyme complex itself or disrupt binding of the holoenzyme to its substrate.

Phosphatases are divided into different classes, including phosphoprotein phosphatases (PPP), metal-dependent protein phosphatases and protein tyrosine phosphatases (PTP). Phosphatases from the PPP class are responsible for the vast majority of dephosphorylations of central nervous system proteins. PPPs, such as PP1 and PP2A class phosphatases, have the characteristic of functioning as a holoenzyme composed of two or more subunits, including a catalytic subunit as well as regulatory subunits. Interestingly, there are few catalytic subunits in the PPP family and the regulatory subunits have therefore generally been thought to confer substrate specificity to the holoenzyme. Emerging structural evidence is supporting this view, namely that phosphatase holoenzymes of the PPP family associate in a structured way and that this structure determines substrate specificity of the dephosphorylation. Some examples are the interaction between PP1 and spinophilin, a complex found in neurons (Ragusa et al., 2010), and the PP1γ–MYPT (myosin phosphatase; PPP1R12A) complex that acts as a myosin phosphatase in muscle (Terrak et al., 2014; Yamashiro et al., 2008). Based on this knowledge, it has been proposed that by targeting the holoenzyme structure, for instance by molecules which disrupt substrate-phosphatase holoenzyme complex, one can specifically modulate phosphorylation levels of proteins (McConnell and Wadzinski, 2009; Tsaytler and Bertolotti, 2013; see conceptual schematic in **Figure 4**). Based on the knowledge of the phosphatases responsible for dephosphorylation of specific phosphosites of proteins, such as emerging knowledge of the composition of phosphatase holoenzymes dephosphorylating specific sites in α-syn, LRRK2, and tau, the specific modulation of phosphorylation levels of single phosphosites is theoretically possible. Further work, including further identification and characterization of specific phosphatase holoenzyme compositions for α-syn, LRRK2, and tau and development of modulators of phosphatase holoenzyme complexes with their specific substrates, is required to test the efficacy of this approach.

## CONCLUSION

The three main proteins linked to Parkinsonism, α-syn, tau, and LRRK2 all display phosphorylations which are modified in disease. The phosphorylation changes are the result of a balance between activity of kinases and phosphatases. Emerging evidence points to phosphatases regulating different pathological phosphorylations in these three proteins, primarily PP1 for LRRK2 and PP2A for α-syn and tau. It appears now feasible to target phosphatases of α-syn, tau, and LRRK2 in order to alleviate pathology mediated by pathological phosphorylation of these disease proteins. In order for this approach to reach its full potential, additional research will be required further linking individual or clustered phosphorylation events in these three proteins to disease. Also, refined knowledge of the precise holoenzyme compositions for each pathological phosphorylation is necessary to develop highly specific phosphatase holoenzyme modulators.

## Conflict of Interest Statement

The authors declare that the research was conducted in the absence of any commercial or financial relationships that could be construed as a potential conflict of interest.

## References

[B1] Anderson J. P. Walker D. E. Goldstein J. M. De Laat R. Banducci K. Caccavello R. J. (2006). Phosphorylation of Ser-129 is the dominant pathological modification of alpha-synuclein in familial and sporadic Lewy body disease. *J. Biol. Chem.* 281 29739–29752 10.1074/jbc.M600933200 16847063

[B2] Bennecib M. Gong C. X. Grundke-Iqbal I. Iqbal K. (2000). Role of protein phosphatase-2A and -1 in the regulation of GSK-3, cdk5, and cdc2 and the phosphorylation of tau in rat forebrain. *FEBS Lett.* 485 87–93 10.1016/S0014-5793(00)02203-1 11086171

[B3] Biernacka J. M. Armasu S. M. Cunningham J. M. Ahlskog J. E. Chung S. J. Maraganore D. M. (2011). Do interactions between SNCA, MAPT, and LRRK2 genes contribute to Parkinson’s disease susceptibility? Parkinsonism *Relat. Disord.* 17 730–736 10.1016/j.parkreldis.2011.07.001 PMC4723425 21816655

[B4] Bollen M. Peti W. Ragusa M. J. Beullens M. (2010). The extended PP1 toolkit: designed to create specificity. *Trends Biochem. Sci.* 35 450–458 10.1016/j.tibs.2010.03.002 20399103 PMC3131691

[B5] Braithwaite S. P. Voronkov M. Stock J. B. Mouradian M. M. (2012). Targeting phosphatases as the next generation of disease modifying therapeutics for Parkinson’s disease. *Neurochem. Int.* 61 899–906 10.1016/j.neuint.2012.01.031 22342821

[B6] Buee L. Bussiere T. Buee-Scherrer V. Delacourte A. Hof P. R. (2000). Tau protein isoforms, phosphorylation and role in neurodegenerative disorders. *Brain Res. Brain Res. Rev.* 33 95–130 10.1016/S0165-0173(00)00019-9 10967355

[B7] Chartier-Harlin M. C. Kachergus J. Roumier C. Mouroux V. Douay X. Lincoln S. (2004). Alpha-synuclein locus duplication as a cause of familial Parkinson’s disease. *Lancet* 364 1167–1169 10.1016/S0140-6736(04)17103-1 15451224

[B8] Chen L. Feany M. B. (2005). Alpha-synuclein phosphorylation controls neurotoxicity and inclusion formation in a *Drosophila* model of Parkinson disease. *Nat. Neurosci.* 8 657–663 10.1038/nn1443 15834418

[B9] Chen L. Periquet M. Wang X. Negro A. Mclean P. J. Hyman B. T. (2009). Tyrosine and serine phosphorylation of alpha-synuclein have opposing effects on neurotoxicity and soluble oligomer formation. *J. Clin. Invest.* 119 3257–3265 10.1172/JCI39088 19855133 PMC2769182

[B10] Chen S. Li B. Grundke-Iqbal I. Iqbal K. (2008). I1PP2A affects tau phosphorylation via association with the catalytic subunit of protein phosphatase 2A. *J. Biol. Chem.* 283 10513–10521 10.1074/jbc.M709852200M709852200 18245083 PMC2447634

[B11] Choi H. G. Zhang J. Deng X. Hatcher J. M. Patricelli M. P. Zhao Z. (2012). Brain penetrant LRRK2 inhibitor. *ACS Med. Chem. Lett.* 3 658–662 10.1021/ml300123a 23066449 PMC3467149

[B12] Daher J. P. Pletnikova O. Biskup S. Musso A. Gellhaar S. Galter D. (2012). Neurodegenerative phenotypes in an A53T alpha-synuclein transgenic mouse model are independent of LRRK2. *Hum. Mol. Genet.* 21 2420–2431 10.1093/hmg/dds057 22357653 PMC3349422

[B13] Daher J. P. Volpicelli-Daley L. A. Blackburn J. P. Moehle M. S. West A. B. (2014). Abrogation of alpha-synuclein-mediated dopaminergic neurodegeneration in LRRK2-deficient rats. *Proc. Natl. Acad. Sci. U.S.A.* 111 9289–9294 10.1073/pnas.14032151111403215111 24927544 PMC4078806

[B14] Delbroek L. Van Kolen K. Steegmans L. Da Cunha R. Mandemakers W. Daneels G. (2013). Development of an enzyme-linked immunosorbent assay for detection of cellular and in vivo LRRK2 S935 phosphorylation. *J. Pharm. Biomed. Anal.* 76 49–58 10.1016/j.jpba.2012.12.002 23313773 PMC4196644

[B15] Del Ser T. Steinwachs K. C. Gertz H. J. Andres M. V. Gomez-Carrillo B. Medina M. (2013). Treatment of Alzheimer’s disease with the GSK-3 inhibitor tideglusib: a pilot study. *J. Alzheimers. Dis.* 33 205–215 10.3233/JAD-2012-120805 22936007

[B16] Deng X. Dzamko N. Prescott A. Davies P. Liu Q. Yang Q. (2011). Characterization of a selective inhibitor of the Parkinson’s disease kinase LRRK2. *Nat. Chem. Biol.* 7 203–205 10.1038/nchembio.538 21378983 PMC3287420

[B17] Doggett E. A. Zhao J. Mork C. N. Hu D. Nichols R. J. (2012). Phosphorylation of LRRK2 serines 955 and 973 is disrupted by Parkinson’s disease mutations and LRRK2 pharmacological inhibition. *J. Neurochem.* 120 37–45 10.1111/j.1471-4159.2011.07537.x 22004453

[B18] Duka V. Lee J. H. Credle J. Wills J. Oaks A. Smolinsky C. (2013). Identification of the sites of tau hyperphosphorylation and activation of tau kinases in synucleinopathies and Alzheimer’s diseases. *PLoS ONE* 8:e75025 10.1371/journal.pone.0075025 PMC3779212 24073234

[B19] Dzamko N. Chua G. Ranola M. Rowe D. B. Halliday G. M. (2013). Measurement of LRRK2 and Ser910/935 phosphorylated LRRK2 in peripheral blood mononuclear cells from idiopathic Parkinson’s disease patients. *J. Parkinsons Dis.* 3 145–152 10.3233/JPD-130174 23938344

[B20] Dzamko N. Deak M. Hentati F. Reith A. D. Prescott A. R. Alessi D. R. (2010). Inhibition of LRRK2 kinase activity leads to dephosphorylation of Ser(910)/Ser(935), disruption of 14-3-3 binding and altered cytoplasmic localization. *Biochem. J.* 430 405–413 10.1042/BJ20100784 20659021 PMC3631100

[B21] Dzamko N. Inesta-Vaquera F. Zhang J. Xie C. Cai H. Arthur S. (2012). The IkappaB kinase family phosphorylates the Parkinson’s disease kinase LRRK2 at Ser935 and Ser910 during Toll-like receptor signaling. *PLoS ONE* 7:e39132 10.1371/journal.pone.0039132 PMC3377608 22723946

[B22] Fujiwara H. Hasegawa M. Dohmae N. Kawashima A. Masliah E. Goldberg M. S. (2002). alpha-Synuclein is phosphorylated in synucleinopathy lesions. *Nat. Cell Biol.* 4 160–164 10.1038/ncb748 11813001

[B23] Gan-Or Z. Bar-Shira A. Mirelman A. Gurevich T. Giladi N. Orr-Urtreger A. (2012). The age at motor symptoms onset in LRRK2-associated Parkinson’s disease is affected by a variation in the MAPT locus: a possible interaction. *J. Mol. Neurosci.* 46 541–544 10.1007/s12031-011-9641-0 21898123

[B24] Gasser T. (2009). Molecular pathogenesis of Parkinson disease: insights from genetic studies. *Expert Rev. Mol. Med.* 11 e22. 10.1017/S1462399409001148 19631006

[B25] Gloeckner C. J. Boldt K. Von Zweydorf F. Helm S. Wiesent L. Sarioglu H. (2010). Phosphopeptide analysis reveals two discrete clusters of phosphorylation in the N-terminus and the Roc domain of the Parkinson-disease associated protein kinase LRRK2. *J. Proteome Res.* 9 1738–1745 10.1021/pr9008578 20108944

[B26] Gong C. X. Liu F. Wu G. Rossie S. Wegiel J. Li L. (2004). Dephosphorylation of microtubule-associated protein tau by protein phosphatase 5. *J. Neurochem.* 88 298–310 10.1111/j.1471-4159.2004.02147.x 14690518

[B27] Greggio E. Bisaglia M. Civiero L. Bubacco L. (2011). Leucine-rich repeat kinase 2 and alpha-synuclein: intersecting pathways in the pathogenesis of Parkinson’s disease? *Mol. Neurodegener.* 6:6 10.1186/1750-1326-6-6 PMC3035023 21244648

[B28] Greggio E. Taymans J. M. Zhen E. Y. Ryder J. Vancraenenbroeck R. Beilina A. (2009). The Parkinson’s disease kinase LRRK2 autophosphorylates its GTPase domain at multiple sites. *Biochem. Biophys. Res. Commun.* 389 449–454 10.1016/j.bbrc.2009.08.163 19733152 PMC2759846

[B29] Guo J. L. Covell D. J. Daniels J. P. Iba M. Stieber A. Zhang B. (2013). Distinct alpha-synuclein strains differentially promote tau inclusions in neurons. *Cell* 154 103–117 10.1016/j.cell.2013.05.057 23827677 PMC3820001

[B30] Hasegawa M. Fujiwara H. Nonaka T. Wakabayashi K. Takahashi H. Lee V. M.-Y. (2002). Phosphorylated alpha-synuclein is ubiquitinated in alpha-synucleinopathy lesions. *J. Biol. Chem.* 277 49071–49076 10.1074/jbc.M208046200 12377775

[B31] Healy D. G. Falchi M. O’Sullivan S. S. Bonifati V. Durr A. Bressman S. (2008). Phenotype, genotype, and worldwide genetic penetrance of LRRK2-associated Parkinson’s disease: a case-control study. *Lancet Neurol.* 7 583–590 10.1016/S1474-4422(08)70117-0 18539534 PMC2832754

[B32] Henderson-Smith A. Chow D. Meechoovet B. Aziz M. Jacobson S. A. Shill H. A. (2013). SMG1 identified as a regulator of Parkinson’s disease-associated alpha-synuclein through siRNA screening. *PLoS ONE* 8:e77711 10.1371/journal.pone.0077711 PMC3813773 24204929

[B33] Herzig M. C. Bidinosti M. Schweizer T. Hafner T. Stemmelen C. Weiss A. (2012). High LRRK2 levels fail to induce or exacerbate neuronal alpha-synucleinopathy in mouse brain. *PLoS ONE* 7:e36581 10.1371/journal.pone.0036581 PMC3352901 22615783

[B34] Houlden H. Singleton A. B. (2012). The genetics and neuropathology of Parkinson’s disease. *Acta Neuropathol.* 124 325–338 10.1007/s00401-012-1013-5 22806825 PMC3589971

[B35] Kahle P. J. Neumann M. Ozmen L. Haass C. (2000). Physiology and pathophysiology of alpha-synuclein. Cell culture and transgenic animal models based on a Parkinson’s disease-associated protein. *Ann. N. Y. Acad. Sci.* 920 33–41 10.1111/j.1749-6632.2000.tb06902.x 11193173

[B36] Kamikawaji S. Ito G. Iwatsubo T. (2009). Identification of the autophosphorylation sites of LRRK2. *Biochemistry* 48 10963–10975 10.1021/bi9011379 19824698

[B37] Kawakami F. Yabata T. Ohta E. Maekawa T. Shimada N. Suzuki M. (2012). LRRK2 phosphorylates tubulin-associated tau but not the free molecule: LRRK2-mediated regulation of the tau-tubulin association and neurite outgrowth. *PLoS ONE* 7:e30834 10.1371/journal.pone.0030834 PMC3267742 22303461

[B38] Kett L. R. Boassa D. Ho C. C. Rideout H. J. Hu J. Terada M. (2012). LRRK2 Parkinson disease mutations enhance its microtubule association. *Hum. Mol. Genet.* 21 890–899 10.1093/hmg/ddr526 22080837 PMC3263991

[B39] Khan N. L. Jain S. Lynch J. M. Pavese N. Abou-Sleiman P. Holton J. L. (2005). Mutations in the gene LRRK2 encoding dardarin (PARK8) cause familial Parkinson’s disease: clinical, pathological, olfactory and functional imaging and genetic data. *Brain* 128 2786–2796 10.1093/brain/awh667 16272164

[B40] Kramer T. Schmidt B. Lo Monte F. (2012). Small-molecule inhibitors of GSK-3: structural insights and their application to Alzheimer’s disease models. *Int. J. Alzheimers Dis.* 2012 381029 10.1155/2012/381029 PMC3408674 22888461

[B41] Kuszczyk M. Gordon-Krajcer W. Lazarewicz J. W. (2009). Homocysteine-induced acute excitotoxicity in cerebellar granule cells in vitro is accompanied by PP2A-mediated dephosphorylation of tau. *Neurochem. Int.* 55 174–180 10.1016/j.neuint.2009.02.010 19428823

[B42] Lambert J. C. Ibrahim-Verbaas C. A. Harold D. Naj A. C. Sims R. Bellenguez C. (2013). Meta-analysis of 74,046 individuals identifies 11 new susceptibility loci for Alzheimer’s disease. *Nat. Genet.* 45 1452–1458 10.1038/ng.2802ng.2802 24162737 PMC3896259

[B43] Lee K. W. Chen W. Junn E. Im J. Y. Grosso H. Sonsalla P. K. (2011). Enhanced phosphatase activity attenuates {alpha}-synucleinopathy in a mouse model. *J. Neurosci.* 31 6963–6971 10.1523/JNEUROSCI.6513-10.2011 21562258 PMC5038983

[B44] Li X. Wang Q. J. Pan N. Lee S. Zhao Y. Chait B. T. (2011). Phosphorylation-dependent 14-3-3 binding to LRRK2 is impaired by common mutations of familial Parkinson’s disease. *PLoS ONE* 6:e17153 10.1371/journal.pone.0017153 PMC3046972 21390248

[B45] Lin C. H. Tsai P. I. Wu R. M. Chien C. T. (2010). LRRK2 G2019S mutation induces dendrite degeneration through mislocalization and phosphorylation of tau by recruiting autoactivated GSK3ss. *J. Neurosci.* 30 13138–13149 10.1523/JNEUROSCI.1737-10.2010 20881132 PMC6633523

[B46] Lin X. Parisiadou L. Gu X. L. Wang L. Shim H. Sun L. (2009). Leucine-rich repeat kinase 2 regulates the progression of neuropathology induced by Parkinson’s-disease-related mutant alpha-synuclein. *Neuron* 64 807–827 10.1016/j.neuron.2009.11.006 20064389 PMC2807409

[B47] Liu F. Grundke-Iqbal I. Iqbal K. Gong C. X. (2005). Contributions of protein phosphatases PP1, PP2A, PP2B and PP5 to the regulation of tau phosphorylation. *Eur. J. Neurosci.* 22 1942–1950 10.1111/j.1460-9568.2005.04391.x 16262633

[B48] Lobbestael E. Baekelandt V. Taymans J. M. (2012). Phosphorylation of LRRK2: from kinase to substrate. *Biochem. Soc. Trans.* 40 1102–1110 10.1042/BST20120128 22988873

[B49] Lobbestael E. Zhao J. Rudenko I. N. Beylina A. Gao F. Wetter J. (2013). Identification of protein phosphatase 1 as a regulator of the LRRK2 phosphorylation cycle. *Biochem. J.* 456 119–128 10.1042/BJ20121772BJ20121772 23937259 PMC5141581

[B50] Lou H. Montoya S. E. Alerte T. N. Wang J. Wu J. Peng X. (2010). Serine 129 phosphorylation reduces the ability of alpha-synuclein to regulate tyrosine hydroxylase and protein phosphatase 2A in vitro and in vivo. *J. Biol. Chem.* 285 17648–17661 10.1074/jbc.M110.100867M110.100867 20356833 PMC2878529

[B51] MacLeod D. Dowman J. Hammond R. Leete T. Inoue K. Abeliovich A. (2006). The familial Parkinsonism gene LRRK2 regulates neurite process morphology. *Neuron* 52 587–593 10.1016/j.neuron.2006.10.008 17114044

[B52] Manning G. Whyte D. B. Martinez R. Hunter T. Sudarsanam S. (2002). The protein kinase complement of the human genome. *Science* 298 1912–1934 10.1126/science.1075762298/5600/1912 12471243

[B53] Martin L. Latypova X. Terro F. (2011). Post-translational modifications of tau protein: implications for Alzheimer’s disease. *Neurochem. Int.* 58 458–471 10.1016/j.neuint.2010.12.023 21215781

[B54] Martin L. Latypova X. Wilson C. M. Magnaudeix A. Perrin M. L. Terro F. (2013a). Tau protein phosphatases in Alzheimer’s disease: the leading role of PP2A. *Ageing Res. Rev.* 12 39–49 10.1016/j.arr.2012.06.008 22771380

[B55] Martin L. Latypova X. Wilson C. M. Magnaudeix A. Perrin M. L. Yardin C. (2013b). Tau protein kinases: involvement in Alzheimer’s disease. *Ageing Res. Rev.* 12 289–309 10.1016/j.arr.2012.06.003 22742992

[B56] Martin L. Magnaudeix A. Esclaire F. Yardin C. Terro F. (2009). Inhibition of glycogen synthase kinase-3beta downregulates total tau proteins in cultured neurons and its reversal by the blockade of protein phosphatase-2A. *Brain Res.* 1252 66–75 10.1016/j.brainres.2008.11.057 19071093

[B57] Mbefo M. K. Paleologou K. E. Boucharaba A. Oueslati A. Schell H. Fournier M. (2010). Phosphorylation of synucleins by members of the Polo-like kinase family. *J. Biol. Chem.* 285 2807–2822 10.1074/jbc.M109.081950 19889641 PMC2807335

[B58] McConnell J. L. Wadzinski B. E. (2009). Targeting protein serine/threonine phosphatases for drug development. *Mol. Pharmacol.* 75 1249–1261 10.1124/mol.108.053140 19299564 PMC2684880

[B59] Melrose H. L. Dachsel J. C. Behrouz B. Lincoln S. J. Yue M. Hinkle K. M. (2010). Impaired dopaminergic neurotransmission and microtubule-associated protein tau alterations in human LRRK2 transgenic mice. *Neurobiol. Dis.* 40 503–517 10.1016/j.nbd.2010.07.010 20659558 PMC2955774

[B60] Moorhead G. B. Trinkle-Mulcahy L. Ulke-Lemee A. (2007). Emerging roles of nuclear protein phosphatases. *Nat. Rev. Mol. Cell Biol.* 8 234–244 10.1038/nrm2126 17318227

[B61] Muda K. Bertinetti D. Gesellchen F. Hermann J. S. Von Zweydorf F. Geerlof A. (2014). Parkinson-related LRRK2 mutation R1441C/G/H impairs PKA phosphorylation of LRRK2 and disrupts its interaction with 14-3-3. *Proc. Natl. Acad. Sci. U.S.A.* 111 E34–E43 10.1073/pnas.13127011111312701111 24351927 PMC3890784

[B62] Nalls M. A. Plagnol V. Hernandez D. G. Sharma M. Sheerin U. M. Saad M. (2011). Imputation of sequence variants for identification of genetic risks for Parkinson’s disease: a meta-analysis of genome-wide association studies. *Lancet* 377 641–649 10.1016/S0140-6736(10)62345-8 21292315 PMC3696507

[B63] Neumann M. Kahle P. J. Giasson B. I. Ozmen L. Borroni E. Spooren W. (2002). Misfolded proteinase K-resistant hyperphosphorylated alpha-synuclein in aged transgenic mice with locomotor deterioration and in human alpha-synucleinopathies. *J. Clin. Invest.* 110 1429–1439 10.1172/JCI200215777 12438441 PMC151810

[B64] Nichols R. J. Dzamko N. Morrice N. A. Campbell D. G. Deak M. Ordureau A. (2010). 14-3-3 binding to LRRK2 is disrupted by multiple Parkinson’s disease-associated mutations and regulates cytoplasmic localization. *Biochem. J.* 430 393–404 10.1042/BJ20100483 20642453 PMC2932554

[B65] Oueslati A. Schneider B. L. Aebischer P. Lashuel H. A. (2013). Polo-like kinase 2 regulates selective autophagic alpha-synuclein clearance and suppresses its toxicity in vivo. *Proc. Natl. Acad. Sci. U.S.A.* 110 E3945–E3954 10.1073/pnas.13099911101309991110 23983262 PMC3799334

[B66] Paleologou K. E. Oueslati A. Shakked G. Rospigliosi C. C. Kim H. Y. Lamberto G. R. (2010). Phosphorylation at S87 is enhanced in synucleinopathies, inhibits alpha-synuclein oligomerization, and influences synuclein-membrane interactions. *J. Neurosci.* 30 3184–3198 10.1523/JNEUROSCI.5922-09.2010 20203178 PMC2947449

[B67] Peng X. Tehranian R. Dietrich P. Stefanis L. Perez R. G. (2005). Alpha-synuclein activation of protein phosphatase 2A reduces tyrosine hydroxylase phosphorylation in dopaminergic cells. *J. Cell Sci.* 118 3523–3530 10.1242/jcs.02481 16030137

[B68] Perez-Revuelta B. I. Hettich M. M. Ciociaro A. Rotermund C. Kahle P. J. Krauss S. (2014). Metformin lowers Ser-129 phosphorylated alpha-synuclein levels via mTOR-dependent protein phosphatase 2A activation. *Cell Death Dis.* 5 e1209. 10.1038/cddis.2014.175cddis2014175 PMC4047877 24810045

[B69] Polymeropoulos M. H. Lavedan C. Leroy E. Ide S. E. Dehejia A. Dutra A. (1997). Mutation in the alpha-synuclein gene identified in families with Parkinson’s disease. *Science* 276 2045–2047 10.1126/science.276.5321.2045 9197268

[B70] Pungaliya P. P. Bai Y. Lipinski K. Anand V. S. Sen S. Brown E. L. (2010). Identification and characterization of a leucine-rich repeat kinase 2 (LRRK2) consensus phosphorylation motif. *PLoS ONE* 5:e13672 10.1371/journal.pone.0013672 PMC2965117 21060682

[B71] Puschmann A. Englund E. Ross O. A. Vilarino-Guell C. Lincoln S. J. Kachergus J. M. (2012). First neuropathological description of a patient with Parkinson’s disease and LRRK2 p.*N1437H* mutation. *Parkinsonism Relat. Disord.* 18 332–338 10.1016/j.parkreldis.2011.11.019 22154298 PMC3330199

[B72] Qing H. Wong W. McGeer E. G. McGeer P. L. (2009). Lrrk2 phosphorylates alpha synuclein at serine 129: Parkinson disease implications. *Biochem. Biophys. Res. Commun.* 387 149–152 10.1016/j.bbrc.2009.06.142 19576176

[B73] Ragusa M. J. Dancheck B. Critton D. A. Nairn A. C. Page R. Peti W. (2010). Spinophilin directs protein phosphatase 1 specificity by blocking substrate binding sites. *Nat. Struct. Mol. Biol.* 17 459–464 10.1038/nsmb.1786 20305656 PMC2924587

[B74] Rahman A. Grundke-Iqbal I. Iqbal K. (2005). Phosphothreonine-212 of Alzheimer abnormally hyperphosphorylated tau is a preferred substrate of protein phosphatase-1. *Neurochem. Res.* 30 277–287 10.1007/s11064-005-2483-9 15895832

[B75] Rahman A. Grundke-Iqbal I. Iqbal K. (2006). PP2B isolated from human brain preferentially dephosphorylates Ser-262 and Ser-396 of the Alzheimer disease abnormally hyperphosphorylated tau. *J. Neural. Transm.* 113 219–230 10.1007/s00702-005-0313-5 15959850

[B76] Reyniers L. Del Giudice M.-G. Civiero L. Belluzzi E. Lobbestael E. Beilina A. (2014). Differential protein-protein interactions of LRRK1 and LRRK2 indicate roles in distinct cellular signaling pathways. *J. Neurochem.* 131 239–250 10.1111/jnc.12798 PMC4272680 24947832

[B77] Reynolds A. Doggett E. A. Riddle S. M. Lebakken C. S. Nichols R. J. (2014). LRRK2 kinase activity and biology are not uniformly predicted by its autophosphorylation and cellular phosphorylation site atatus. *Front. Mol. Neurosci.* 7:54 10.3389/fnmol.2014.00054 PMC4068021 25009464

[B78] Saito T. Ishiguro K. Uchida T. Miyamoto E. Kishimoto T. Hisanaga S. (1995). In situ dephosphorylation of tau by protein phosphatase 2A and 2B in fetal rat primary cultured neurons. *FEBS Lett.* 376 238–242 10.1016/0014-5793(95)01292-0 7498550

[B79] Satake W. Nakabayashi Y. Mizuta I. Hirota Y. Ito C. Kubo M. (2009). Genome-wide association study identifies common variants at four loci as genetic risk factors for Parkinson’s disease. *Nat. Genet.* 41 1303–1307 10.1038/ng.485 19915576

[B80] Sharma M. Ioannidis J. P. Aasly J. O. Annesi G. Brice A. Van Broeckhoven C. (2012). Large-scale replication and heterogeneity in Parkinson disease genetic loci. *Neurology* 79 659–667 10.1212/WNL.0b013e318264e353 22786590 PMC3414661

[B81] Sheng Z. Zhang S. Bustos D. Kleinheinz T. Le Pichon C. E. Dominguez S. L. (2012). Ser1292 autophosphorylation is an indicator of LRRK2 kinase activity and contributes to the cellular effects of PD mutations. *Sci. Transl. Med.* 4 164ra161. 10.1126/scitranslmed.30044854/164/164ra161 23241745

[B82] Simon-Sanchez J. Schulte C. Bras J. M. Sharma M. Gibbs J. R. Berg D. (2009). Genome-wide association study reveals genetic risk underlying Parkinson’s disease. *Nat. Genet.* 41 1308–1312 10.1038/ng.487 19915575 PMC2787725

[B83] Singleton A. B. Farrer M. Johnson J. Singleton A. Hague S. Kachergus J. (2003). alpha-Synuclein locus triplication causes Parkinson’s disease. *Science* 302 841 10.1126/science.1090278302/5646/841 14593171

[B84] Sontag E. Luangpirom A. Hladik C. Mudrak I. Ogris E. Speciale S. (2004). Altered expression levels of the protein phosphatase 2A ABalphaC enzyme are associated with Alzheimer disease pathology. *J. Neuropathol. Exp. Neurol.* 63 287–301. 15099019 10.1093/jnen/63.4.287

[B85] Sontag E. Nunbhakdi-Craig V. Lee G. Brandt R. Kamibayashi C. Kuret J. (1999). Molecular interactions among protein phosphatase 2A, tau, and microtubules. Implications for the regulation of tau phosphorylation and the development of tauopathies. *J.* *Biol. Chem.* 274 25490–25498 10.1074/jbc.274.36.25490 10464280

[B86] Spillantini M. G. Schmidt M. L. Lee V. M. Trojanowski J. Q. Jakes R. Goedert M. (1997). Alpha-synuclein in Lewy bodies. *Nature* 388 839–840 10.1038/42166 9278044

[B87] Takahashi M. Kanuka H. Fujiwara H. Koyama A. Hasegawa M. Miura M. (2003). Phosphorylation of alpha-synuclein characteristic of synucleinopathy lesions is recapitulated in alpha-synuclein transgenic *Drosophila*. *Neurosci. Lett.* 336 155–158 10.1016/S0304-3940(02)01258-2 12505616

[B88] Taymans J. M. Cookson M. (2010). Mechanisms of dominant parkinsonism; the toxic triangle of LRRK2, alpha-synuclein and tau. *Bioessays* 32 227–235 10.1002/bies.200900163 20127702 PMC4662284

[B89] Tell V. Hilgeroth A. (2013). Recent developments of protein kinase inhibitors as potential AD therapeutics. *Front. Cell. Neurosci.* 7 189 10.3389/fncel.2013.00189 PMC3832900 24312003

[B90] Tenreiro S. Eckermann K. Outeiro T. F. (2014). Protein phosphorylation in neurodegeneration: friend or foe? *Front. Mol. Neurosci.* 7 42 10.3389/fnmol.2014.00042 PMC4026737 24860424

[B91] Terrak M. Kerff F. Langsetmo K. Tao T. Dominguez R. (2004). Structural basis of protein phosphatase 1 regulation. *Nature* 429 780–784 10.1038/nature02582 15164081

[B92] Tsaytler P. Bertolotti A. (2013). Exploiting the selectivity of protein phosphatase 1 for pharmacological intervention. *FEBS J.* 280 766–770 10.1111/j.1742-4658.2012.08535.x 22340633

[B93] Ujiie S. Hatano T. Kubo S. Imai S. Sato S. Uchihara T. (2012). LRRK2 I2020T mutation is associated with tau pathology. *Parkinsonism Relat. Disord.* 18 819–823 10.1016/j.parkreldis.2012.03.024 22525366

[B94] Vancraenenbroeck R. Lobbestael E. De Maeyer M. Baekelandt V. Taymans J. M. (2011). Kinases as targets for Parkinson’s disease; from genetics to therapy. *CNS Neurol. Disord. Drug Targets* 10 724–740 10.2174/187152711797247858 21838679

[B95] Vancraenenbroeck R. Lobbestael E. Weeks S. D. Strelkov S. V. Baekelandt V. Taymans J. M. (2012). Expression, purification and preliminary biochemical and structural characterization of the leucine rich repeat namesake domain of leucine rich repeat kinase 2. *Biochim. Biophys. Acta* 1824 450–460 10.1016/j.bbapap.2011.12.009 22251894

[B96] Virshup D. M. Shenolikar S. (2009). From promiscuity to precision: protein phosphatases get a makeover. *Mol. Cell.* 33 537–545 10.1016/j.molcel.2009.02.015 19285938

[B97] Voronkov M. Braithwaite S. P. Stock J. B. (2011). Phosphoprotein phosphatase 2A: a novel druggable target for Alzheimer’s disease. *Future Med. Chem.* 3 821–833 10.4155/fmc.11.47 21644827 PMC3292348

[B98] Walker I. Newell H. (2009). Do molecularly targeted agents in oncology have reduced attrition rates? *Nat. Rev. Drug Discov.* 8 15–16 10.1038/nrd2758 19008887

[B99] Waxman E. A. Giasson B. I. (2008). Specificity and regulation of casein kinase-mediated phosphorylation of alpha-synuclein. *J. Neuropathol. Exp. Neurol.* 67 402–416 10.1097/NEN.0b013e31816fc995 18451726 PMC2930078

[B100] Waxman E. A. Giasson B. I. (2011). Characterization of kinases involved in the phosphorylation of aggregated alpha-synuclein. *J. Neurosci. Res.* 89 231–247 10.1002/jnr.22537 21162130 PMC4484797

[B101] Webber P. J. Smith A. D. Sen S. Renfrow M. B. Mobley J. A. West A. B. (2011). Autophosphorylation in the leucine-rich repeat kinase 2 (LRRK2) GTPase domain modifies kinase and GTP-binding activities. *J. Mol. Biol.* 412 94–110 10.1016/j.jmb.2011.07.033 21806997 PMC3158845

[B102] West A. B. Moore D. J. Choi C. Andrabi S. A. Li X. Dikeman D. (2007). Parkinson’s disease-associated mutations in LRRK2 link enhanced GTP-binding and kinase activities to neuronal toxicity. *Hum. Mol. Genet.* 16 223–232 10.1093/hmg/ddl471 17200152

[B103] Xu Y. Chen Y. Zhang P. Jeffrey P. D. Shi Y. (2008). Structure of a protein phosphatase 2A holoenzyme: insights into B55-mediated Tau dephosphorylation. *Mol. Cell.* 31 873–885 10.1016/j.molcel.2008.08.006 18922469 PMC2818795

[B104] Yamashiro S. Yamakita Y. Totsukawa G. Goto H. Kaibuchi K. Ito M. (2008). Myosin phosphatase-targeting subunit 1 regulates mitosis by antagonizing polo-like kinase 1. *Dev. Cell* 14 787–797 10.1016/j.devcel.2008.02.013 18477460 PMC2680213

[B105] Zhang J. Deng X. Choi H. G. Alessi D. R. Gray N. S. (2012). Characterization of TAE684 as a potent LRRK2 kinase inhibitor. *Bioorg. Med. Chem. Lett.* 22 1864–1869 10.1016/j.bmcl.2012.01.084 22335897 PMC3433743

[B106] Zimprich A. Biskup S. Leitner P. Lichtner P. Farrer M. Lincoln S. (2004). Mutations in LRRK2 cause autosomal-dominant parkinsonism with pleomorphic pathology. *Neuron* 44 601–607 10.1016/j.neuron.2004.11.005 15541309

